# Report of the Diseases of Edinburgh for July, 1810

**Published:** 1810-09

**Authors:** John Roberton


### *Report of the Diseases of Edinburgh for July,* 1810. By John Roberton, M. D.

The weather, during the early part of the month, was wet and disagreeable, and, after sun-set, the fogs which enveloped the whole city were dense and excessively unpleasant. Toward the middle of the month, however, the weather became somewhat more settled, and we had some delightful days. With few exceptions, and these only of very short duration, the weather continued pleasant till the termination of the month.

The barometer, although rather low during the first half of the month, was not so much so as on former occasions, even when the falls of rain were less heavy. After this it rose considerably, and seemed scarcely at all altered by the slight changes of weather, and, when the month terminated, it still rose high.

The thermometer stood high during the whole month, except, indeed, during a few of the foggy nights, when it sunk even below 40°. The general range of it was, however, from about 60 to 70.

The

The Diseases, although not in general of a very formidable nature, have been greater in number than during last month. The wet weather about the beginning of the month seems to have given origin to various cases of severe catarrh, to cynanche tonsillaris, to rheumatism, and diarrhœa. Croup has also appeared in various parts of the city and neighbourhood. Measles and chin-cough, which had almost entirely disappeared, have been met with in practice; and fevers, which are never entirely absent, have been of the very worst kind.

Catarrh prevailed very universally, and of almost every degree of severity. In various cases, the lungs have even been slightly affected with inflammation; and in two cases the patients, being evidently predisposed to phthisis pulmonalis, had that disease induced from the above cause. Patients predisposed to this formidable and fatal complaint, are often impressed with those flattering prospects so commonly entertained by phthisical patients, and too often not only inadvertently expose themselves to the probability of its attack, but, as was the case in both the patients alluded to, decline administering any thing for the relief of the catarrh till phthisical symptoms supervened, when, according to our present very imperfect knowledge of these matters, it was too late.

Cynanche tonsillaris has also been very universal in its attack, and in many cases very severe. Although in most instances purgative medicines, with gargles of the diluted acids, have in general effected its complete removal, yet in many cases these means had very little or no effect in arresting the symptoms. In addition to them, the repeated application of blisters over the whole fore part of the throat was of singular benefit. Such was the severity of several cases which came under my observation, that the submaxillary glands seemed greatly enlarged, and in a few instances they even suppurated, and broke externally. This not only proved exceedingly troublesome at the time, but the after consequences of such a state were very unpleasant. These appearances gave origin to the supposition of scrofula existing in the system, when no such disease was present. This ought at all times to stimulate us to the very utmost exertion to prevent such unpleasant consequences. We should not rest contented with having used low diet, purgatives, and blisters, but, in addition to them, we ought to employ general and topical blood-letting, which is frequently followed by the most happy effects. I think I have observed, when the inflammatory action action had not proceeded to suppuration, that the frequent application of warm emollient poultices to the throat, and even the daily use of the warm bath, were, in addition to the above means, of eminent service.

Rheumatism, both chronic and acute, have been occasionally met with in practice. These I have taken notice of in my former reports with what seemed to me the most rational method of alleviating or removing them, and to these I shall now refer my readers.

Diarrhœa is by no means an uncommon disease in this place about this time of the year. It seems principally caused by the quantity of unripe fruits, with which this part of the world abounds, and which are profusely used by a great many people. The dampness in the early parts of the month too, probably contributed to its presence. In the first of these, purgative medicines remove it in a very short time, but in some instances I have even found it necessary previously to administer an emetic. In the latter, although purgative medicines may often be used with advantage, yet, as the existence of the complaint did not seem so immediately owing to an accumulation of foreign matter in the intestinal canal, they were not, in every case, so immediately necessary. In some cases, indeed, the disease evidently existed from a sort of irritation in the intestines, which purgatives tended more to increase than allay. In such, I have found hyosciamus with a small proportion of opium, of the greatest benefit. It is only by attending to particular existing circumstances, in these as well as in every other complaint, that we are to expect decided success. Not by the plan too common in practice, of entirely overlooking the delicate structure and admirable mechanism of the human body; and rather treating it as a chemist's crucible, than with that minuteness of attention which its very nature requires. By the one method, we reduce ourselves to mere mechanics of the most dangerous sort, but by the other, we are entitled to rank high in a useful, an honourable, but still very imperfect profession.

Croup has been more commonly felt among us, than I recollect to have seen it for several years. Yet although it has, in some cases, terminated fatally, it has in general been rather of a mild form. Sub-muriate of mercury of course has been the principle remedy we have employed, and in general it has answered our expectations.

The measles and chin-cough, both of which had left us for several weeks, again appeared in various parts of the the city. None of the cases, however, have been very severe.

Fevers of the very worst and most contagious kind, have appeared in the low and ill-ventilated parts of the Old Town; these have even spread to other parts, and have committed such ravages, as are inseparable from the existence of such a disease.

*Princes Street.*

